# Dengue Fever in the Darfur Area, Western Sudan

**DOI:** 10.3201/eid2511.181766

**Published:** 2019-11

**Authors:** Ayman Ahmed, Yousif Ali, Babiker Elmagboul, Omaima Mohamed, Adel Elduma, Hind Bashab, Ahmed Mahamoud, Hayat Khogali, Arwa Elaagip, Tarig Higazi

**Affiliations:** Liverpool School of Tropical Medicine, Liverpool, UK (A. Ahmed);; University of Khartoum, Khartoum, Sudan (A. Ahmed, A. Elaagip);; Sudan Federal Ministry of Health, Khartoum (Y. Ali, B. Elmagboul, O. Mohamed, A. Elduma, H. Bashab, A. Mahamoud, H. Khogali);; Ohio University, Zanesville, Ohio, USA (T. Higazi)

**Keywords:** dengue, dengue virus, viruses, outbreak, emergence, serotypes, dengue shock syndrome, dengue hemorrhagic fever, epidemic, arbovirus, zoonoses, Darfur, Sudan

## Abstract

We report an outbreak of dengue in Darfur, western Sudan, during September 2014–April 2015. Dengue virus–specific PCR testing of 50 samples from nonmalaria febrile illness case-patients confirmed 35 dengue cases. We detected 7 cases of dengue shock syndrome and 24 cases of dengue hemorrhagic fever.

Dengue is a mosquito-transmitted arboviral disease caused by 4 closely related dengue virus serotypes (DENV1–4); the primary vector of DENV is *Aedes aegypti* mosquitoes (*1*). Dengue infection has different clinical manifestations of disease, ranging from a self-limiting illness to the fatal severe forms of dengue hemorrhagic fever or dengue shock syndrome (*1*).

Dengue is a rapidly growing global public health problem, and cases have been identified in >128 countries (*2*,*3*). Several factors might contribute to dengue transmission, including human population growth, density, and movement, and international travel and trade (*4*,*5*), as well as scarcity and poor storage of water and global climate change. Secondary infection with different virus serotypes and sex and young age of patients seem to be associated with development of severe disease (*1*).

The Darfur region of Sudan is composed of 5 states covering an area of 493,180 km^2^ of desert and semidesert. This region has a population of 7.5 million persons living in a humanitarian crisis since 2003. We report dengue fever in this region of western Sudan.

On September 16, 2014, a large number of case-patients with nonmalarial febrile illness came to outpatient clinics in AlFashir, the capital of North Darfur State. An outbreak investigation team was assembled and deployed to the area by the Federal Ministry of Health. Using active surveillance, this team identified 155 suspected cases of hemorrhagic fever in various localities within the state through April 12, 2015. The suspected case-patients had fever (152/155), bleeding (140/155), headache (73/155), joint pain (52/155), and neurologic signs (9/155). Most case-patients came from AlFashir, the initial location of the outbreak. The outbreak peaked during October 2014 with 77 (49.7%) suspected cases (Figure). Most (52%) of the suspected case-patients were <20 years of age (52%) (age range 18 months–74 years), and the female:male ratio was 1:1.5.

**Figure:**
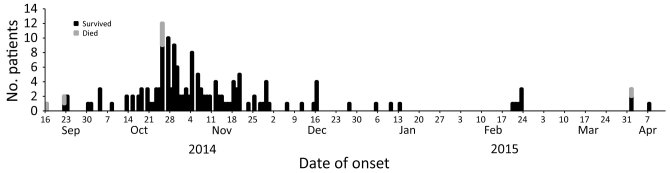
Number of dengue case-patients per week, Darfur area, western Sudan, September 16, 2014–April 7, 2015.

Clinical manifestations of the suspected case-patients suggested involvement of hemorrhagic fevers. Considering the history of similar epidemics of fevers in Sudan and our limited resources, we tested samples for yellow fever virus, Rift Valley fever virus, and DENV (*6*,*7*). We tested blood samples from suspected case-patients for these infections by using IgM-specific ELISAs, and all positive results were confirmed by use of a disease-specific PCR in the Central National Public Health Laboratory in Khartoum (*7*).

We obtained only 50 blood samples from 155 suspected case-patients because of patient or family refusal to participate in this study. A total of 35 (70.0%) samples were positive for DENV-1 or DENV-3 serotypes. Eight deaths occurred among the DENV-positive persons (mortality rate 5.2%) during the investigation period. Four of the fatal cases were in children <14 years of age, 2 in adult men, and 2 in adult women. Clinical examination of the confirmed infected persons identified 7 cases of dengue shock syndrome and 24 cases of dengue hemorrhagic fever.

We report emergence of dengue in the Greater Darfur area of Sudan. Dengue is a major public health issue in this country, but had been confined to the eastern region of the country and the Red Sea coastal and subcoastal states (*4*). Frequent outbreaks have been reported to the World Health Organization Regional Office for the Eastern Mediterranean (*6*). Greater Darfur, a region affected by a civil war, has had massive population displacement, resulting in most persons living in densely populated refugee camps with limited access to education and health services. Lack of water supply necessitated its storage in human-made containers (*8*), which favored breeding of *Ae. aegypti* mosquitoes and increased human–mosquito contact.

The severity of these infections could be because our surveillance selected only the most severe cases, enhanced by the poor healthcare-seeking behavior of the local population, who came to health clinics only when the disease was severe (*7*). The actual prevalence of dengue could be much higher than that detected because our surveillance system likely detected only the most severe cases (*7*). A wider and better surveillance system is urgently needed to detect nonsevere cases and determine the actual prevalence of the disease and population at risk.

In addition, Gayer et al. suggested that socioeconomic and environmental changes associated with the civil war in Sudan made communities vulnerable to the emergence of infectious diseases (*8*). The geopolitical and security issues surrounding the refugee camps suggest that dengue was imported into the area through members of United Nations Peacekeeping Forces from dengue-endemic areas rather than being introduced from East Sudan, as has been observed in Australia (*8*–*10*).

Our study had some limitations. First, we observed weak healthcare-seeking behavior, which resulted in insufficient blood samples for diagnosis. Second, because of limited resources, we could not investigate co-infections with DENV-1 and DENV-3. Third, our survey was conducted in healthcare clinics where only severe case-patients were seen. We recommend improvement of surveillance and development of an early warning system to reduce future impacts of such epidemics. We also highlight the need to improve health and living conditions of persons living in humanitarian crisis settings.
